# Enhancing Commercial Gourmet Oil Quality: The Role of Dried Cayenne Pepper Red (*Capsicum annuum* L.) as a Natural Additive

**DOI:** 10.3390/molecules30040927

**Published:** 2025-02-17

**Authors:** Zuzana Knazicka, Branislav Galik, Ivana Novotna, Julius Arvay, Katarina Fatrcova-Sramkova, Miroslava Kacaniova, Jiri Mlcek, Eva Kovacikova, Eva Mixtajova, Tunde Jurikova, Eva Ivanisova, Adriana Kolesarova, Hana Duranova

**Affiliations:** 1 Institute of Nutrition and Genomics, Faculty of Agrobiology and Food Resources, Slovak University of Agriculture, Trieda Andreja Hlinku 2, 949 76 Nitra, Slovakia; zuzana.knazicka@uniag.sk (Z.K.); branislav.galik@uniag.sk (B.G.); ivana.novotna@uniag.sk (I.N.); katarina.sramkova@uniag.sk (K.F.-S.); eva.kovacikova@uniag.sk (E.K.); 2 Institute of Food Sciences, Faculty of Biotechnology and Food Sciences, Slovak University of Agriculture, Trieda Andreja Hlinku 2, 949 76 Nitra, Slovakia; julius.arvay@uniag.sk (J.A.); eva.ivanisova@uniag.sk (E.I.); 3 Institute of Horticulture, Faculty of Horticulture and Landscape Engineering, Slovak University of Agriculture, Trieda Andreja Hlinku 2, 949 76 Nitra, Slovakia; miroslava.kacaniova@uniag.sk; 4 School of Medical and Health Sciences, University of Economics and Human Sciences in Warsaw, Okopowa 59, 010 43 Warsaw, Poland; 5 Department of Food Analysis and Chemistry, Faculty of Technology, Tomas Bata University in Zlin, Vavreckova 5669, 760 01 Zlin, Czech Republic; mlcek@utb.cz; 6 Faculty of Agrobiology and Food Resources, Slovak University of Agriculture, Trieda Andreja Hlinku 2, 949 76 Nitra, Slovakia; eva.mixtajova@uniag.sk; 7 Institute for Teacher Training, Faculty of Central European Studies, Constantine the Philosopher University in Nitra, Drazovska 4, 949 74 Nitra, Slovakia; tjurikova@ukf.sk; 8 Institute of Applied Biology, Faculty of Biotechnology and Food Sciences, Slovak University of Agriculture, Trieda Andreja Hlinku 2, 949 76 Nitra, Slovakia; adriana.kolesarova@uniag.sk; 9 AgroBioTech Research Centre, Slovak University of Agriculture, Trieda Andreja Hlinku 2, 949 76 Nitra, Slovakia

**Keywords:** vegetable oils, Cayenne pepper red, fatty acids, technological profile, antioxidant activity, antimicrobial activity, health benefits

## Abstract

This study assessed the potential of dried Cayenne pepper (CP; *Capsicum annuum* L.) as a natural additive to rice bran oil (RBO), grape seed oil (GSO), and virgin olive oil (OO). Key analyses included peroxide and acid values, oxidative stability (Rancimat method), the composition of fatty acids (FAs) (GC-FID method), antioxidant activity (AA; DPPH method), and antimicrobial properties (disc diffusion method). Capsaicin and the dihydrocapsaicin contents in CP were quantified (HPLC-DAD method) as 1499.37 ± 3.64 and 1449.04 ± 5.14 mg/kg DW, respectively. Oleic acid (C18:1cis n9) dominated in OO (69.70%), OO-CP (69.73%), and RBO-CP (38.97%), while linoleic acid (C18:2cis n6) prevailed in RBO (41.34%), GSO (57.93%), and GSO-CP (58.03%). The addition of CP influenced the FA profile, particularly linoleic acid in OO and RBO, and all FAs in GSO. Peroxide and acid values increased significantly in RBO and GSO upon CP addition, but induction times remained unaffected. The strongest AA (77.00 ± 0.13%) was observed in OO-CP. Cayenne pepper significantly enhanced the antioxidant profiles of all oils compared to the counterparts. However, the antimicrobial activity was weak (≤5.0 mm inhibition zones) against tested microorganisms. These findings support CP as a functional additive for enhancing the nutritional and functional properties of gourmet oils, while highlighting the need for further optimization to improve stability and bioactivity.

## 1. Introduction

With the population and economic development increasing worldwide, the public is becoming more concerned about the health benefits and nutritional properties of unconventional vegetable oils [1]. The current trend involves utilizing food industry by-products like rice bran oil (RBO), which is derived from rice production [2]. RBO is a popular cooking oil in Japan and India, and its popularity is rapidly growing in other Asian countries, where it is regarded as a “healthy oil”. The oil has gained significant attention due to its high quality, extended shelf life, and its favorable fatty acid (FA) profile [3], which includes oleic acid (C18:1; 36–45%), linoleic acid (C18:2; 30–38%), and palmitic acid (C16:0; 15–22%) [4,5]. While RBO contains only a small proportion of α-linolenic acid (C18:3 n-3), it is sufficient for the de novo synthesis of other n-3 polyunsaturated fatty acids (PUFAs), particularly eicosapentaenoic acid (EPA; C20:5 n-3) and docosahexaenoic acid (DHA; C22:6 n-3), in tissue phospholipids. Additionally, RBO is rich in phytonutrients like phytosterols, γ-oryzanol, squalene, triterpene alcohols, and vitamin E derivatives (tocopherols and tocotrienols) [2]. These components provide high antioxidant [6], anti-inflammatory [7], hypocholesterolemic, antidiabetic [2,8], and anticancer properties [9,10]. Some studies have suggested that RBO possesses biological activities that may help prevent colorectal cancer [11]. Yu et al. [10] specifically found that RBO inhibits the cancer cell cycle, promotes apoptosis, and enhances chemopreventive effects, making it a promising adjuvant in cancer prevention and treatment. Additionally, the dietary intake of RBO has been linked to reduced levels of low-density lipoprotein (LDL), total cholesterol, and blood pressure, as well as reduced inflammation and the alleviation of metabolic syndrome symptoms [2,12,13]. Furthermore, RBO supports immune function and may aid in preventing premature aging and age-related neurodegenerative diseases [6,14].

The cultivation and processing of grapes produce enormous quantities of residues such as grape marc, grape seed, grape skins, grape stems, and grape leaves. These by-products are abundant in phenolic compounds, which have garnered attention in recent research due to their physiological importance [15,16]. Among them, grape seed oil (GSO) has emerged as a particularly valuable functional food. This oil is rich in hydrophilic constituents, such as flavonoids, carotenoids, phenolic acids, tannins, and stilbenes [17], as well as lipophilic compounds like vitamins E and C, FAs, and phytosterols [18]. The composition and amount of these bioactive compounds can vary depending on the grape variety, harvest conditions, and oil extraction methods [19,20]. In addition, GSO typically contains 58–78% linoleic acid, 14–25% oleic acid, 7.0–8.0% palmitic acid, and <0.60% linolenic acid, though the exact composition may differ based on grape type [21,22]. Its soft fruity taste, high smoke point (210–230 °C), minimal viscosity increase during frying, and high digestibility further emphasize GSO’s value as a versatile and healthful oil [23], suitable for both infants and the elderly [24,25]. The appealing organoleptic properties of GSO, including its pleasant aroma and flavor, have contributed to its growing use in the culinary world [20]. Beside this, it has demonstrated potential in pharmacological and cosmetic applications [26,27]. Numerous studies have highlighted its health-promoting properties, such as anti-inflammatory, cardioprotective, antimicrobial, antiatherogenic, antithrombotic, hypolipidemic, and anticancer effects [28,29]. Phytosterols, in particular, play a crucial biological role, contributing to antioxidant activity and cholesterol metabolism [19,20].

Olive oil, extracted from the fruit of *Olea europaea* L. through purely mechanical processes without the use of harsh treatments, stands out among vegetable oils [30]. While it accounts for only 2.0% of the global oil and fat production by volume, it contributes 11.20% of the market value due to its distinctive organoleptic, chemical, and physical qualities. These characteristics make virgin olive oil (OO) a true fruit juice, which must be carefully handled from harvest through processing and storage to maintain its quality [31,32]. Virgin olive oil is highly valued for its nutritional and health benefits, particularly in reducing the risk of cardiovascular disorders. This is attributed to its high levels of monounsaturated fats (MUFAs), mainly oleic acid (55–83%), and the presence of important minor components such as polyphenols (e.g., oleuropein, hydroxytyrosol), phytosterols, tocopherols, carotenoids, chlorophyll, and squalene [33,34,35]. Other key FAs present in olive oil include linoleic acid (2.50–21%), palmitic acid (7.50–20%), and α-linolenic acid (≤1.0%), contributing to its health-promoting profile [35].

Recently, “flavored oils” have gained popularity in the food market [36], especially in non-Mediterranean countries [37]. The addition of various spices and herbs to oils, a long-standing culinary tradition to enhance flavor, has led to the development of “gourmet oils” infused with ingredients like oregano, garlic, basil, and chili peppers (*Capsicum* spp.) [38]. These natural additives [39] aim not only to improve sensory characteristics but also to enhance the nutritional value and extend the shelf life of the oils [40,41]. Among these, *Capsicum* spp. stand out due to their rich composition of bioactive compounds [42], which vary across different pepper types [43]. The genus *Capsicum*, part of the Solanaceae family, consists of 33 species, 10 varieties, and 5 domesticated types: *Capsicum* (*C*.) *baccatum* L., *C*. *chinenses* Jacqs., *C*. *frutescens* L., *C*. *pubescens* Ruiz & Pav., and *C*. *annuum* L., all native to Central and South America [44]. Common varieties include bell peppers, cayenne, jalapeños, and tabasco [45], all of which are known for their distinct flavors and functional properties. Chili peppers are rich in essential micro- and macronutrients [46,47], offering high antioxidant potential and antimicrobial activity. Their fruits contain bioactive compounds such as crude fat, carotenoids, capsaicinoids, proteins, volatile oil, and vitamins, particularly C [43], E, and provitamin A [46]. Additionally, they are abundant in phenolic compounds, including anthocyanins and flavonoids [48] like quercetin, luteolin, and kaempferol [49]. These bioactives are linked to antihypercholesterolemic properties, improved lipid metabolism [50], cancer prevention, a reduced risk of diabetes mellitus, cardiovascular diseases, and obesity [51,52]. Capsaicin and dihydrocapsaicin, the principal capsaicinoids, also exhibit strong neurological, antimutagenic, antitumor, and antioxidant effects [42]. In Cayenne pepper (CP; *Capsicum annuum* L.), these two major capsaicinoids account for 79% to 90% of the total capsaicinoid content, depending on the fruit ripening stage [53]. Compared to other spices with well-documented antioxidant and antimicrobial properties, such as turmeric [54], cinnamon [55], garlic [56] or rosemary [57], CP offers a distinctive combination of pungency and bioactive compounds, including polyphenols and carotenoids [58]. This unique profile not only enhances health benefits but also contributes to oxidative stability [59,60] and improved sensory attributes in oil [61], making them particularly attractive for gourmet applications. Beyond this sensory impact, capsaicin has been linked to digestive benefits, such as stimulating enzyme secretion and aiding digestion [42,62]. Moreover, chili peppers were selected for this study not only for their well-established functional properties but also due to their significant global relevance and market potential. Their widespread use in numerous national cuisines is evident in the substantial rise in annual production over the past two decades—an increase of more than 100%, from 2 million to 4.5 million tons of dried biomass and from 17 million to 36 million tons of fresh biomass [63]. This remarkable growth underscores their increasing consumer demand and commercial significance, making them particularly relevant for enhancing the nutritional and functional properties of edible oils. The primary objective of the present study was to evaluate the impact of dried CP on the key technological properties of three commercial gourmet oils, specifically RBO, GSO, and virgin OO. These oils were selected based on their distinct chemical compositions and functional properties, which may interact differently with CP bioactive compounds. In effect, RBO was used as an oil with a well-balanced ratio of MUFAs and PUFAs [64], GSO as a PUFA-rich oil [20], and virgin OO as a MUFA-dominant oil [35]. Accordingly, this study aimed to determine the potential of CP as a natural additive in food products and to assess how variations in lipid matrices and bioactive compositions across different oils influence the functionality of CP-derived compounds. To achieve this, the analysis focused on several critical aspects, including peroxide and acid values, oxidative stability, and FA composition in both CP-flavored and non-flavored oils. Additionally, the antioxidant capacity and antimicrobial activity of these oils were evaluated to further investigate their potential health benefits and preservative properties.

## 2. Results and Discussion

The global rise in edible oil consumption is constrained by various factors, including the presence of trans-FAs, high susceptibility to oxidation, and undesirable rancid characteristics [65]. To combat these issues, the addition of food additives has become a common practice in food processing. These additives help prevent spoilage, enhance food quality, and improve sensory attributes in a cost-effective manner [66,67]. Currently, consumers are increasingly favoring natural additives over synthetic ones, which also improve food freshness, color, flavor, texture, and nutritional value [68]. This shift in preferences is driven by a growing awareness of health concerns associated with artificial substances [69]. Chili peppers, which are widely produced on a large scale, offer significant advantages in this context. In addition to their well-known health benefits due to their capsaicinoid content, their utilization in various applications also provides opportunities for reducing agricultural waste [70].

### 2.1. Quantitation of Capsaicinoids in Dried Cayenne Pepper Red

Commonly, capsaicinoids are responsible for the pungency and unique taste of pepper cultivars [71], with the capsaicin content being a major parameter in determining their commercial quality [72,73]. Capsaicin also contributes to the pharmaceutical properties of peppers [74]. The amount of capsaicinoids depends on the plant’s genetic makeup and environmental factors [71], such as light intensity, temperature, fruit age, and position on the plant [75]. Additionally, capsaicin levels can vary among different varieties of the same species and even within a single fruit [76].

**Figure 1:**
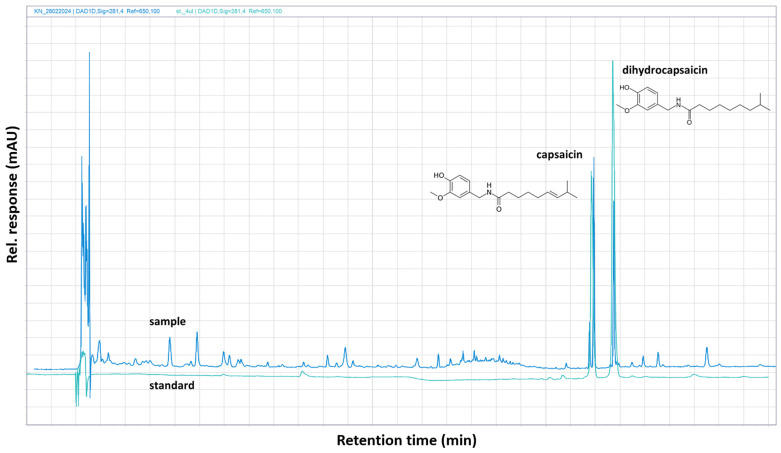
Chromatogram of the standards of capsaicin, dihydrocapsaicin (green peaks), and sample (blue peaks) in dried Cayenne pepper red (*Capsicum annuum* L.). UV detections of capsaicin and dihydrocapsaicin were recorded at a wavelength of 281 nm, retention times of 22.63 min (capsaicin)–23.44 min (dihydrocapsaicin), and using C18 column (150 mm × 3.50 mm × 2.70 µm). Mobile phase was 0.10% phosphoric acid in ddH_2_O (ultra-pure and sterile water) and acetonitrile (HPLC gradient grade).

In our study, the principal capsaicinoids, capsaicin and dihydrocapsaicin, were identified and quantified using HPLC-DAD. In all chromatograms obtained for the analyzed CP, the prominent peaks corresponded to these two primary capsaicinoids. Their ultraviolet absorption spectra were recorded with a photodiode array detector, as shown in Figure 1. The mean concentration of capsaicin in CP was determined to be 1499.37 ± 3.64 mg/kg DW, while that of dihydrocapsaicin was 1449.04 ± 5.14 mg/kg DW. In a study by Lopez-Hernandez et al. [77], CP samples had mean capsaicin and dihydrocapsaicin contents of 1320 and 830 μg/g DW, respectively. Regular consumption of foods containing capsaicinoids and secoiridoids reduces the risk of cardiovascular disease, as reported by [78]. When capsaicinoids from chilies are combined with a high-quality oil containing high levels of secoiridoids and other health-benefiting compounds, the resulting “chili” oil can greatly benefit human health [79].

### 2.2. Evaluation of the Fatty Acid Profiles for Health Benefits

**Figure 2:**
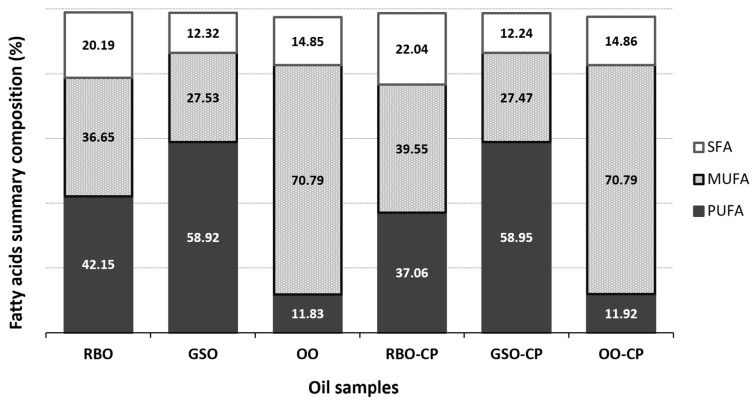
Vegetable oil assessment from the viewpoint of the fatty acid summary composition (%). RBO—rice bran oil; GSO—grape seed oil; OO—virgin olive oil; RBO-CP—rice bran oil flavored with dried Cayenne pepper red; GSO-CP—grape seed oil flavored with dried Cayenne pepper red; OO-CP—virgin olive oil flavored with dried Cayenne pepper red; SFA—saturated fatty acid; MUFA—monounsaturated fatty acid; PUFA—polyunsaturated fatty acid.

The FA composition of the studied oils is presented in Table 1. Oleic acid (C18:1cis n9) was the predominant FA in OO/OO-CP, contributing to the highest MUFA content (70.79%), as illustrated in Figure 2. This lipid fraction is known for its protective properties against coronary, autoimmune, and inflammatory disorders, as well as its anti-thrombotic effects and role in regulating blood pressure [80]. Palmitic acid (C16:0) was the second most important FA in these samples, with values of 11.39% for OO and 11.41% for OO-CP. It was followed by linoleic acid (C18:2cis n6), which ranged from 11.04% in OO to 11.14% in OO-CP. The content of other FAs was notably lower in both oils, which guarantees their longer shelf life and clarity at low temperatures. From a comprehensive overall assessment, no major differences were observed in the FA composition between the flavored OO-CP and non-flavored OO, except for linoleic acid which was demonstrably (*p* < 0.05) higher in OO-CP. The positive impact of OO on health has been previously investigated, showing a correlation between oxidative stress and many diseases such as cardiovascular problems and atherosclerosis [80].

In RBO, the major FAs were linoleic acid (41.34%), oleic acid (36.16%), and palmitic acid (16.74%) (Table 1). Among the oils investigated, RBO/RBO-CP (20.19% vs. 22.04%) (Figure 2) exhibited the highest proportion of SFAs, aligning with the findings of Maszewska et al. [81]. Moreover, these oils were the only ones in which myristic acid (C14:0) was detected, with concentrations ranging from 0.25% (RBO) to 0.31% (RBO-CP). This FA is known for accumulating fat in the body; however, its consumption also impacts positively on cardiovascular health [82]. The addition of CP to RBO contributed to a significant increase in the levels of almost all FAs investigated, most notably (*p* < 0.001) in oleic acid (38.97%) and palmitic acid (18.12%). On the other hand, a considerably (*p* < 0.001) higher concentration of linoleic acid (41.34%) was noted in the RBO sample compared to the RBO-CP sample (36.23%). Both evaluated oils (RBO/RBO-CP) also contained a trace amount of lignoceric acid (C24:0), which demonstrably (*p* < 0.001) differed between each other. The significantly higher levels of nearly all analyzed FAs in RBO-CP compared to its counterpart suggest that CP provides protective effects against the oxidation and degradation of these specific FAs. However, this protective action appears to be selective, as evidenced by the demonstrable (*p* < 0.001) decrease in linoleic acid content in RBO-CP.

Grape seed oil is notably rich in unsaturated FAs, comprising 85–90% of its composition [83]. In line with this, the average content of these acids in both of our oils (GSO or GSO-CP) was approximately 86% (Figure 2). These oils shared the highest amounts of PUFAs among all those analyzed, with 58.92% in GSO and 58.95% in GSO-CP. From a nutritional perspective, the presence of PUFAs in oils is advantageous; however, oils with this composition are less resistant to external factors and are more prone to oxidation (e.g., α-linolenic acid) [84]. Table 1 lists the nine different FAs detected and quantified in the GSO/GSO-CP samples, with linoleic acid (C18:2cis n6) being the predominant FA in both GSO (57.93%) and GSO-CP (58.03%), achieving the highest concentration among all evaluated oils. Demirtas et al. [85] reported that the PUFA content in various native Turkish grape seed oils ranged from 56.65% to 68.97%, with linoleic acid concentrations varying between 56.38% and 68.56%. Wen et al. [86] found that seed oils from grapes cultivated in China exhibited PUFA values ranging from 63.88% to 77.12%, and linoleic acid contents between 63.52% and 76.77%. Oleic acid (C18:1cis n9) was the second most important FA in our samples studied. The minor FAs included stearic acid (C18:0), palmitoleic acid (C16:1), α-linolenic acid (C18:3 n3), arachidic acid (C20:0), cis-11-eicosenoic acid (C20:1 n9), and behenic acid (C22:0).

Very few vegetable oils contain *α*-linolenic acid, which must be supplemented in the diet to safeguard health [87]. Among all our vegetable oils evaluated, the highest content of α-linolenic acid was measured in GSO (0.99%), followed by GSO-CP (0.92%). This also implies a high ∑ n6/n3 FA ratio in these oils, accounting for 58.42 in GSO and 63.09 in GSO-CP. The essential FAs in GSO exert a complex influence on the concentrations of lipoproteins, the fluidity of biological membranes, the function of membrane enzymes and receptors, the modulation of eicosanoid production, blood pressure regulation, the metabolism of minerals [28], and total cholesterol [88,89]. Nash [90] observed that the consumption of up to 45 g/day of GSO increased HDL cholesterol by 13.0% and reduced LDL cholesterol levels by 7.0% in humans. In animal models, however, there are conflicting results regarding changes in serum, muscular, and hepatic lipid profiles after the use of GSO [15,91,92]. Well-designed, randomized clinical trials are needed to evaluate the effects of GSO on the lipid profile in humans.

Interestingly, our study demonstrated a distinct effect of CP addition on the linoleic acid content in RBO, GSO, and OO. This observation can be attributed to several factors related to their composition, as well as the interactions between the CP and the specific FAs and antioxidant systems present in these oils. Consistent with our findings, both GSO (70%) and RBO (33%) have relatively high levels of linoleic acid when compared to OO (10%) [93], which is richer in oleic acid, comprising 55–83% of the total FA content [94]. The significantly higher levels of linoleic acid (*p* < 0.05) found in our CP-flavored GSO and OO may suggest that linoleic acid from CP contributed to the increased concentrations in these flavored oils, or it may indicate a specific interaction between CP and linoleic acid in these oils. In effect, linoleic acid was identified as the major compound (36.60–43.70%) in the pericarp of red pepper (*C*. *annuum* L.) [95], and the seeds of the fruit were shown to be exceptionally rich in linoleic acid (72%) [96], both of which were utilized in our study. Regarding the second hypothesis, linoleic acid is one of the PUFAs, which are major substrates for oil oxidation due to their unsaturated bond having a nature that generates different metabolites along with reactive oxygen species [97]. Thus, this acid is more prone to oxidation compared to other FAs. For instance, the oxidation rates of stearic acid, oleic acid, and linoleic acid have been shown to be 1:100:1200 [98,99]. Hence, linoleic acid could specifically interact with certain components of CP, and in particular, helping its stabilization. The presence of protective antioxidants contained in CP could selectively slow its oxidation, resulting in a higher content of this acid in the OO-CP and GSO-CP compared to their counterparts. Other FAs, which are more stable, may not have been significantly influenced by this protective mechanism, explaining why their levels remained similar between these flavored and non-flavored oils. However, the selective synergistic effects between the oil antioxidants and CP compounds were not effective in GSO-CP for certain specific FAs like palmitic acid (*p* < 0.01) and α-linolenic acid (*p* < 0.001), which levels were found to be significantly lower than in GSO. This fact may suggest that the synergistic machinery has already been diminished in GSO-CP, leading to insufficient protection for these FAs and failing to compensate for their susceptibility to oxidation. Importantly, unlike the remaining flavored oils (GSO-CP and OO-CP), RBO-CP exhibited demonstrably lower linoleic acid content compared to the non-flavored oil. This reduction may be due to negative interactions between CP components, especially capsaicinoids, and the oil’s unique antioxidants, such as γ-oryzanol, which could have accelerated linoleic acid oxidation. Oryzanol, a well-known component in RBO, has a saturated ring system [100], allowing it to function as a strong antioxidant, protecting RBO from oxidation by scavenging free radicals [101]. Based on the chemical structures and antioxidant roles of capsaicinoids and γ-oryzanol, some speculative connections can be highlighted. The main process, responsible for the peroxyl scavenging activity of capsaicin, was found to be the hydrogen transfer from the OH phenolic group [102]. The hydroxyl moiety as the functional group responsible for the antioxidant activity of capsaicinoid in canola oil was also suggested by Si et al. [59]. On the other hand, the antioxidant ability of γ-oryzanol is mainly ascribed to the ferulic moiety [103,104]. In addition, the intramolecular hydrogen bonding in γ-oryzanol, caused by the methoxy group, reduces its ability to scavenge free radicals. The strong electron-withdrawing effect of the methoxy group also increases the oxygen–hydrogen bond strength in the hydroxyl group, further decreasing its efficiency compared to more reactive antioxidants like α-tocopherol [105]. Taking into account these aspects, it can be hypothesized that capsaicinoids could potentially either interfere with the antioxidant mechanism of γ-oryzanol or compete with it for the reaction with free radicals, thereby reducing the reaction capacity of γ-oryzanol with active oxygen. As a result, this antagonistic interaction could diminish the protective effects of γ-oryzanol, leading to increased oxidation of linoleic acid in RBO-CP. To support this hypothesis, capsaicinoids present in CP might enhance the antioxidant stability of our GSO and OO due to the lack of γ-oryzanol. Our speculation is further supported by the findings of Liu et al. [105], who discovered that the combination of α-tocopherol and γ-oryzanol demonstrated significant antagonistic effects at different concentrations. This could be attributed to the similarity in their antioxidant capacities within that concentration range. When combined, they likely compete to react with peroxyl radicals, thereby reducing the ability of α-tocopherol to neutralize reactive oxygen species. Consequently, unsaturated FAs become more prone to oxidation. Anyway, both oryzanol [106,107] and capsaicinoids [108,109] play a role in modulating the oxidative stability of vegetable oils; however, to the best of our knowledge, there is no study examining their combined effects on the stability and oxidative behavior of flavored oils. To explore this potential connection further, future studies would need to assess how the two compounds interact under specific conditions, particularly within oil matrices or food products where both are present. Furthermore, changes in FA composition were observed only in RBO following CP addition, with a reduction in PUFAs accompanied by an increase in MUFAs and SFAs (Figure 2). These alterations are likely linked to the interactions discussed earlier. Anyway, while this shift in FA profile may enhance oxidative stability and extend shelf life, it could also have a negative impact on nutritional value of the oil by decreasing the availability of essential PUFAs.

### 2.3. Evaluation of the Technological Properties of Oil Quality—Peroxide and Acid Values, Oxidative Stability

The peroxide value analysis primarily determines the content of hydroperoxides and peroxides, which are unstable compounds that can break down into aldehydes and ketones [110,111]. This quality parameter is crucial because oxygen contributes to lipoxygenase cascade reactions, affecting positive sensory properties. Indeed, its excess can lead to negative defects [112] like off-flavors and overall deterioration in oil quality. High susceptibility to oxidation limits the use of such oils in the food industry, while a low peroxide value indicates strong oxidative stability and better oil preservation [113]. Importantly, all edible commercial non-flavored oils tested in our study (Table 2) were within the acceptable limit for refined oils (10 mEq O_2_/kg) according to Slovak legislation [114]. The peroxide values for RBO, GSO, and OO were 7.20 ± 0.00, 9.20 ± 0.00, and 9.00 ± 0.28 mEq O_2_/kg, respectively. Comparatively, Pimpa et al. [115] reported that the peroxide value of RBO ranged from 6.59 ± 0.34 to 8.18 ± 0.22 mEq O_2_/kg (0–8 weeks), with a respective increase over the storage period. Similarly, Pardo et al. [116] found that the peroxide value for GSO, depending on the grape variety and extraction method, varied between 5.99 and 13.50 mEq O_2_/kg. On the other hand, virgin OO analyzed in the research by Osanloo et al. [117] showed higher peroxide values, ranging from 10.21 ± 0.01 to 36.72 ± 0.17 mEq O_2_/kg over a 21-day incubation period, compared to the values observed in our OO samples. With the exception of OO, the addition of CP to the other two oils resulted in a significant increase in their peroxide values (Table 2), with RBO-CP showing a 1.6-fold increase and GSO-CP showing a 1.2-fold increase compared to their counterparts. This finding indicates a greater formation of primary oxidation products in RBO-CP and GSO-CP which is in accordance with higher oxidation of specific FAs, detected in these oils (Table 1). Besides the hypothesis mentioned before, this observation could be associated with the pro-oxidant effect of some antioxidants on the peroxide formation in these oils due to their elevated amounts resulting from the mixture of these oils with CP. This suggests that vegetable oils containing lipophilic compounds derived from plant sources may exhibit pro-oxidative properties depending on the oxidation condition, as noted in the study by Yi and Kim [118]. In accordance with our findings, catechin demonstrated a pro-oxidant effect on peroxide formation in sardine oil, despite its high scavenging activity, as proposed in the study by Vaisali et al. [119]. Although the peroxide values of our RBO-CP and GSO-CP were slightly above the limit for refined oils according to Slovak legislation [114], they were still compliant with the standards set by the European Commission (EC) Regulation No. 1989/2003 [120], which states that the peroxide value should not exceed 20 mEq O_2_/kg in cold-pressed oil. This requirement was met in all samples tested in our study. Interestingly, the CP incorporation into OO had no significant effect on the peroxide value, suggesting that CP did not reduce or accelerate the initial stages of oxidation in this oil, and thus did not influence its freshness or stability in terms of oxidation.

The acid value is another important indicator of the oil quality, especially regarding its storage [87,121]. Oxidative and chemical changes in the oil are characterized by an increase in acid value, which results from an increase in the free FA content of the oil. This parameter indicates the amount of acidic substances produced by the oxidation of hydrocarbons, meaning that the acid value directly reflects the degree of oil degradation [113]. In our study, acid values of tested oils ranged from 0.17 ± 0.08 for GSO to 1.40 ± 0.24 mg KOH/g for RBO-CP (Table 2). Evaluation of the non-flavored oils revealed that all vegetable oils had values below 0.60 mg KOH/g, confirming the high quality of the oils and compliance with the strict standards for refined fats and oils according to Slovak legislation [114]. Among the CP-flavored oils, the highest acid values were observed in RBO-CP (1.40 ± 0.24 mg KOH/g) and GSO-CP (1.29 ± 0.24 mg KOH/g) which, like the peroxide values, were significantly different from their counterparts. This fact suggests that some antioxidants present in CP could act as mild pro-oxidative agents, increasing not only peroxide levels but also acid values. In effect, the effectiveness of antioxidants in lipid systems is strongly influenced by multiple factors, including the type of lipid, the hydrophilic–lipophilic balance of the antioxidant, and interfacial interactions. Consequently, an antioxidant that performs well in one system may not exhibit the same efficacy in another [119,122,123]. Principally, these values were within the limit established by the AOCS [124] for oils produced using cold-pressing technology (4.0 mg KOH/g). Similar to the peroxide value, the CP addition to OO did not induce any demonstrable alteration in the acid value of this oil, which is consistent with our data obtained from the analysis of the oil’s FA composition (Table 1).

Conducting the Rancimat test allowed us to determine the induction times of the tested oils (Table 2). From the results, it is evident that OO (9.10 h) and OO-CP (8.38 h), which contain the highest content of MUFAs (70.79%) and the smallest amounts of PUFAs (11.83% and 11.92%, respectively), exhibited the highest oxidation stability among the oils investigated. In general, OO is well-known for its high resistance to oxidative deterioration due to both a triacylglycerol composition low in PUFAs and a group of phenolic antioxidants composed mainly of polyphenols and tocopherols [125]. The oxidative stability of OO depends not only on characteristics such as variety and quality but also on agricultural practices (e.g., cultivation area, harvesting time), as well as the degree of unsaturation and the content of antioxidants, such as tocopherols, phenols, and carotenes. Additionally, the type of extraction process [22] and storage conditions can also significantly affect its conservation [126]. On the other hand, the more unsaturated and the less saturated a fat is, the faster the oxidation reaction proceeds [127,128]. In accordance with this statement, the induction times for our RBO and RBO-CP, displaying a higher content of PUFAs and a lower content of MUFAs, were significantly lower than those of OO and OO-CP. However, they were similar, 5.60 h (for RBO) and 5.21 h (for RBO-CP), when compared to each other. Finally, this statement explains why our GSO (3.48 h) and GSO-CP (3.00 h), characterized by the highest amount of PUFAs (58.92% and 58.95%, respectively, Figure 2) oxidized the fastest among all oils analyzed. Interestingly, CP addition did not demonstrably influence the induction times of any type of oil analyzed, despite the observed changes in peroxide and acid values. This increase in peroxide and acid values could be due to interactions between the oil and certain compounds in the pepper, such as phenolics or minor lipid components, which may influence the oxidative reactions in a different manner. To further support this reasoning, Zellama et al. [37] observed a higher peroxide value along with a longer induction time (measured by Rancimat) in extra virgin OO flavored with chili pepper, indicating that the initial increase in oxidation markers does not necessarily correlate with reduced oxidative stability.

### 2.4. Evaluation of the Antioxidant Activity

**Figure 3:**
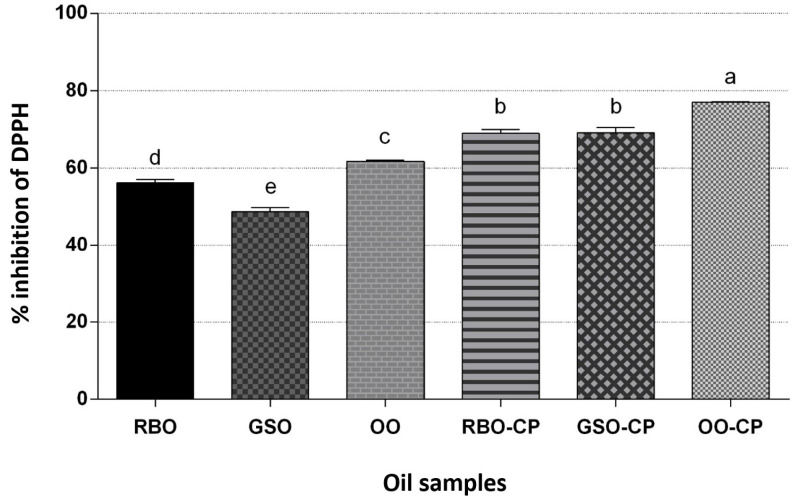
The antioxidant activity of evaluated edible vegetable oils. RBO—rice bran oil; GSO—grape seed oil; OO—virgin olive oil; RBO-CP—rice bran oil flavored with dried Cayenne pepper red; GSO-CP—grape seed oil flavored with dried Cayenne pepper red; OO-CP—virgin olive oil flavored with dried Cayenne pepper red. Each bar represents the arithmetic mean (±standard deviation). The power of antioxidant activity was assessed based on the following scheme: weak (0–29%) < medium-strong (30–59%) < strong (60% and more); different small letters indicate statistically significant differences at the level *p* < 0.05 between oils.

Given that oil oxidation can occur through various pathways, catalyzed by different initiators such as peroxides, trace metals, and photosensitizers, it is essential to analyze the in vitro antioxidant capacity to function as a radical scavenger [119]. When comparing the antioxidant activity (DPPH method) of each type of oil, we observed considerable differences. The lowest antioxidant activity was measured for GSO (48.63 ± 1.54%). Fernandes et al. [26] reported values for DPPH radical scavenging activities of various GSOs ranging from 38.68% to 69.89%, depending on the grape variety. Xia et al. [129] compared the antioxidant capacity of grapes and their by-products. The highest antioxidant capacity, measured by the oxygen radical absorbance capacity assay, was found in grape seeds (42.18 mmol TE/g). In the present study, the addition of CP to all oils significantly increased their antioxidant profiles compared to the control, non-flavored commercial oils (Figure 3). The strongest antioxidant activity was measured for OO-CP (77.00 ± 0.13%), which can be a reason for the preservation of its peroxide and acidic values, alongside its oxidative stability. Similarly, a study by Caporaso et al. [130] reported that the highest antioxidant activity was found in olive oil with a 20% addition of chili peppers. Our results can be attributed to the increased content of antioxidants (e.g., carotenoids, capsaicinoids, phenolic compounds, ascorbic acid, etc.) or the concentrations of reducing compounds due to the addition of CP, thereby enhancing the protective properties against free radicals. On the other hand, the addition of CP had the strongest impact in terms of elevated radical scavenging ability in GSO. This fact can be hypothetically connected with the significantly decreased content of palmitic acid and α-linolenic acid, as well as demonstrably higher peroxide and acid values in GSO-CP, assuming the action of elevated antioxidants as pro-oxidants, initiating oxidative reactions rather than preventing them. This can paradoxically reduce the oil’s oxidative stability instead of improving it. In line with this hypothesis, Vaisali et al. [119] reported a pro-oxidant effect of catechin on peroxide formation in sardine oil, despite its strong scavenging activity. Additionally, no significant differences in antioxidant capacity have been identified between GSO-CP and RBO-CP oils, which also showed significantly decreased content of linoleic acid, peroxide, and acid values, as well as changes in the overall FA composition, further supporting this hypothesis. Thus, strong radical-scavenging ability is beneficial up to a point; however, an overactive or imbalanced antioxidant response can sometimes destabilize the oil, leading to premature oxidation or a reduction in overall shelf life. Therefore, the balance of antioxidant capacity and oxidative stability is crucial.

To gain a comprehensive understanding of the mechanisms driving the increased antioxidant activity, further analysis is warranted. This should include the quantification of specific bioactive compounds transferred into the oil, the assessment of their individual and synergistic antioxidant effects, and the evaluation of how different oil matrices modulate these interactions. Such studies would elucidate whether the observed enhancement in antioxidant activity is primarily due to the concentration of bioactive compounds or specific interactions within the oil matrix.

### 2.5. Evaluation of the Antimicrobial Activity

The results on the antimicrobial activity of six vegetable oils tested by the disc diffusion method are shown in Table 3. Based on the zone of inhibition, all oils were classified as having weak (≤5.0 mm) antimicrobial activity against the tested microorganisms. In RBO, the weakest antimicrobial activity was found against *Candida tropicalis* (1.33 mm), while the best activity was determined against *Listeria monocytogenes* (2.67 mm). *Pseudo-monas aeruginosa*, *Candida glabrata*, and *Candida tropicalis* were the most resistant microorganisms (1.33 mm) against the inhibitory action of RBO-CP, while *Staphylococcus aureus* subsp. *aureus*, *Bacillus cereus*, and *Salmonella enterica* subsp. *enterica* showed the most sensitivity (2.33 mm) to this oil. The most potent antibacterial activity of OO was observed against *Staphylococcus aureus* subsp. *aureus* (2.67 mm), *Listeria monocytogenes* (2.67 mm), *Bacillus cereus* (2.67 mm), and *Pseudomonas aeruginosa* (2.67 mm). Based on the results, it can be seen that the flavored OO with CP was the most effective, mainly against the growth of *Salmonella enterica* subsp. *enterica* (2.67 mm). The results by Zellama et al. [37] showed that the flavored extra virgin OO with dried chili pepper (*Capsicum annuum* L.) of the Chemsi F1 variety was more active on Gram-positive bacteria. This oil was most active against the *Bacillus cereus* strain. In the present study, the inhibition zone of GSO ranged from 1.67 mm (*Listeria monocytogenes*, *Candida albicans*, *Candida krusei*, and *Candida tropicalis*) to 2.67 mm (*Salmonella enterica* subsp. *enterica*). Similar weak antibacterial activity, with a maximum zone of inhibition of 2.67 mm, was also determined for GSO-CP against *Salmonella enterica* subsp. *enterica* and *Yersinia enterocolitica*. Khah et al. [131] determined the antibacterial properties of different, active emulsified films containing GSO (0.15 g/g biopolymer). These active films showed antibacterial effects against *Salmonella typhimurium* (12.10 mm), *Staphylococcus aureus* (10.0 mm), *Pseudomonas fluorescence* (11.06 mm), and *Escherichia coli* 157:H7 (11.03 mm).

The antioxidant and antimicrobial activity of chili pepper varieties are influenced by the presence of individual biologically active substances, as previously described [132]. Capsaicinoids appeared to inhibit fungi and both Gram-positive/negative bacterial growth in several studies. In all of them, capsaicin/dihydrocapsaicin showed a significant degree of inhibition, stopping or slowing colony development [133,134]. Some interesting results of antimicrobial tests with capsaicinoids reported that they inhibited the growth of *Escherichia coli*, *Listeria monocytogenes*, *Bacillus subtillis*, and many strains of *Staphylococcus aureus* [135]. The results of Kumral and ve Şahin [136] showed that water extracts of Cayenne pepper inhibited the presence of *Enterobacter aerogenes* and *Listeria monocytogenes*. In a study by Omolo et al. [137], researchers explored the relationship between the content of capsaicin (1.737–42.633 µg/mL) and the antimicrobial activity of chili pepper extracts from 29 unexplored varieties of *Capsicum* against *Listeria monocytogenes*, *Staphylococcus aureus*, *Salmonella typhimurium*, *Escherichia coli* O157:H7, and *Candida albicans*. The findings revealed that the antimicrobial activity of pepper extracts did not consistently increase with higher capsaicin concentrations, suggesting that various *Capsicum* cultivars may possess distinct capsaicin derivatives. Regarding the pathogens, *Listeria monocytogenes* and *Staphylococcus aureus* were more vulnerable to the antimicrobial effects of capsaicin than *Salmonella typhimurium* and *Escherichia coli*. *Candida albicans* was markedly more susceptible to the impact of capsaicin than all bacterial species tested.

## 3. Materials and Methods

### 3.1. Chemicals

All the chemicals used were of analytical grade and purchased from either Sigma-Aldrich (St. Louis, MO, USA), CentralChem (Bratislava, Slovakia), or Supelco (St. Louis, MO, USA).

### 3.2. Materials

The material comprised six types of commercially available edible oils: rice bran oil (RBO), grape seed oil (GSO), virgin olive oil (OO), and these oils flavored with whole dried Cayenne pepper red (CP; *Capsicum annuum* L.) fruits, including the placenta, seeds, and pericarp. This resulted in the production of RBO-CP, GSO-CP, and OO-CP. The samples of all oils investigated were purchased from a specialty store in Nitra (Slovakia), from a local producer that also utilizes various chili pepper varieties including CP. The samples of oils were stored in the dark at a low temperature (10 °C) with daily shaking.

### 3.3. Determination of Capsaicinoids in Cayenne Pepper Red (Capsicum annuum L.) by HPLC-DAD Method

The major capsaicinoids (capsaicin and dihydrocapsaicin) in dried CP were determined using high-performance liquid chromatography—HPLC (Agilent 1260 Infinity II; Agilent Technologies GmbH, Wäldbronn, Germany). The dried samples of CP were crushed using an IKA A10 grinder (IKA-Werke GmbH & Co. KG, Staufen, Germany) and subsequently extracted with 20 mL of 80% ethanol (*v*/*v*) at laboratory temperature for 2 h using a shaker Unimax 2010 (Heidolph Instruments GmbH, Germany). The extract was filtered through Munktell No. 390 paper (Munktell & Filtrak GmbH, Bärenstein, Germany) and stored in closed 50 mL PE tubes. Prior to HPLC analysis, the extract was filtered through a syringe filter QMax (0.22 µm, 25 mm, PVDF) (Frisenette ApS, Knebel, Denmark). All HPLC separations were performed on a Cortecs^®^ reverse phase C18 column (150 mm × 3.50 mm × 2.70 µm) (Waters Inc., Milford, MA, USA), using gradient elution with a mobile phase comprising 0.10% phosphoric acid in ddH_2_O (ultra-pure and sterile water) and acetonitrile (HPLC gradient grade) [138]. Capsaicin and dihydrocapsaicin, purchased from Sigma-Aldrich (St. Louis, MO, USA), were certified ACS grade with more than 95% purity. The detection wavelengths of capsaicin and dihydrocapsaicin were set at 281 nm. The analytical method was validated by determining the limit of detection (LOD) and the limit of quantification (LOQ) for both capsaicinoids. The LODs of the proposed method were 12.30 μg/g for capsaicin and 7.69 μg/g for dihydrocapsaicin, while the LOQs were 40.10 μg/g and 23.10 μg/g, respectively. Data were collected and processed using Agilent OpenLab CDS 2.3—chromatography. The concentrations of capsaicinoids in CP samples were expressed in mg/kg dry weight (DW).

### 3.4. Determination of Fatty Acid Composition by GC-FID Method

Gas chromatography (GC) with a flame ionization detector (FID) on an Agilent 6890A (Agilent Technologies, Santa Clara, CA, USA) with a multi-mode injector was used to quantify FAs. Fatty acids were separated on a DB-23 analytical column (Agilent Technologies 122-2361; 60 m × 250 µm × 0.15 µm). For column calibration, a 37-component standard Supelco mixture (Supelco 47885-U; Sigma-Aldrich, Laramie, WY, USA) was used. A total of 200 mg of the sample was used in a 20 mL test tube with a ground joint neck. The sample was dissolved in 5.0 mL of n-hexane, and 1.0 mL of 2N KOH in methanol was added. This was followed by intensive shaking of the test tube, which was then placed in a water bath heated to 60 °C for 30 s. After shaking, the tubes were left to rest for 1 min. Subsequently, 2.0 mL of hydrochloric acid (33–36% p.a. HCl) was added, and the contents were gently shaken. Once equilibrium was reached and the layers separated, the upper layer containing FAs was carefully pipetted out, filtered through anhydrous Na_2_SO_4_, and used for GC-FID analysis. The esterified oil samples were then diluted at a ratio of 1:19 (50 µL FA + 950 µL n-hexane) before analysis. The elution time of the separated analytes served as a quality indicator. Chromatograms of the samples were compared with the standard chromatogram. Conversely, the area under the peak of the monitored analyte was the indicator of quantity. All analytical gases used (He, H_2_, N_2_, and synthetic air) had a purity of 5.0. The information was processed online with Agilent OpenLab ChemStation software (OpenLab CDS ChemStation Edition B.04.01).

### 3.5. Technological Properties of Oils Quality

#### 3.5.1. Determination of Peroxide Value

The peroxide value measures the amount of hydroperoxides formed through oxidation during storage, expressed as milliequivalents (mEq) of active oxygen per kilogram of oil (mEq O_2_/kg). The determination of the peroxide value was conducted as follows: a 5.0 g sample of oil was dissolved in 100 mL of a chloroform/acetic acid mixture (2:3, *v*/*v*) and allowed to react with potassium iodide solution in the dark. Subsequently, the free iodine was titrated with a 0.01 N sodium thiosulfate solution [87]. This analysis followed the analytical methods described by AOCS [139]. The peroxide value was calculated using the following Formula (1):

(1) $${\mathrm{Peroxide}\;\mathrm{value}\;(\mathrm{mEq}\;O}_{2}/\mathrm{kg})\;=\frac{1000\times \left(a\;-b\right)\times \;N}{m}$$

where 1000—the conversion factor; N—normality of the sodium thiosulfate solution; a—the volume of sodium thiosulfate solution used in the blank titration (mL); b—the volume of the sodium thiosulfate solution used in the sample titration (mL); and m—the mass of the oil sample (g).

#### 3.5.2. Determination of Acid Value

The procedure for the determination of the acid value was taken from a publication by Ivanišová et al. [87]. Into an Erlenmeyer flask, 5.0 g of the sample was weighed. For each sample of oil, 100 mL of a mixture containing equal volumes of ethanol and chloroform (1:1) was prepared. The prepared solution was gently heated to boiling in a hot bath (GFL 1013; Zevenhuizen, The Netherlands). After mixing, 2–3 drops of phenolphthalein indicator were added, and the contents were titrated with 0.1 M KOH solution under hot and continuous stirring until a pinkish color persisted for 30 s [140]. The result was expressed as the number of milligrams of KOH required to neutralize the free FAs in 1.0 g of the sample (mg KOH/g). The acid value was calculated using the following formula [124]:

(2) $$\mathrm{Acid}\;\mathrm{value}\;\left(\mathrm{mg}\;\mathrm{KOH}/g\right)=\frac{V\times C\;\times 56.10}{m}$$

where V—the volume of the titration KOH solution consumed (mL); C—the concentration of the KOH solution (mol/L); m—the mass of the oil (g); and 56.10—the molar mass of potassium hydroxide (g/mol).

#### 3.5.3. Determination of Oxidative Stability by Rancimat Method

The Rancimat method is based on an accelerated oxidation process and the formation of volatile compounds, achieved by exposing samples to elevated temperatures and simultaneous air injection. The measurement typically spans several hours, in contrast to the weeks or months needed for other conventional methods. This method simulates an accelerated aging process during which volatile oxidation products are transferred by a stream of air to a vessel and absorbed into the measuring solution (distilled water). The conductivity of the solution is consistently monitored throughout the experiment. A sample of 3.0 g of oil was used to determine the oxidative stability in the Rancimat 892 apparatus from Metrohm (Zofingen, Switzerland) [87], which operates and records the entire measurement process through the StabNet software compatible with model 892. The experiments were conducted at a temperature of 120 °C with a steady airflow of 20 L/h. The results are presented as software-generated curves displaying the induction time in hours.

### 3.6. Determination of Antioxidant Activity by DPPH Method

The antioxidant activity of oils was determined based on the free radical scavenging capacity using the stable 2,2-diphenyl-1-picrylhydrazyl radical (DPPH•). Procedure for the determination of antioxidant activity was taken from a publication by Ivanišová et al. [87]. Briefly, 0.40 mL of the samples was mixed with 3.60 mL of a DPPH• solution (0.025 g of DPPH• dissolved in 100 mL of ethanol). After resting for 10 min in the dark at room temperature, the absorbance was measured at 515 nm with a Jenway 6405 UV/VIS spectrophotometer (London, UK). The percentage of inhibition of DPPH was calculated from Equation (3), as follows:

(3) $$\%\;\mathrm{Inhibition}\;\mathrm{of}\;\mathrm{DPPH}=\frac{A_{0}-\;\mathrm{AA}\;}{A_{0}}\times \;100$$

where A_0_—the absorbance of the DPPH• in solution without an antioxidant, and AA—the absorbance of the DPPH• solution in the presence of an antioxidant. Trolox (6-hydroxy-2,5,7,8-tetramethylchroman-2-carboxylic acid; Sigma-Aldrich, Schnelldorf, Germany; concentration range 10–100 mg/L; R^2^ = 0.9881) was used as the standard reference substance. The power of antioxidant activity was assessed based on the following scheme: weak (0–29%) < medium-strong (30–59%) < strong (60% and more).

### 3.7. Determination of Antimicrobial Activity by Disc Diffusion Method

The effectiveness of the antimicrobial activity was assessed using the disc diffusion method, which is used to determine the susceptibility of microbial strains to antimicrobial agents. Depending on the size of the inhibitory zones formed around disks soaked in the antimicrobial on the solid agar, the strains can be divided into sensitive or resistant ones. However, the disc diffusion method does not determine the degree of sensitivity of individual strains, but rather whether the microorganisms are sensitive or resistant to selected test substances. For the testing, a Petri dish filled with Mueller–Hinton agar (MHA; Oxoid, Basingstoke, UK) was used for each bacterial strain, while a Petri dish filled with Sabouraud’s dextrose agar (SDA; Oxoid, Basingstoke, UK) was used for the yeast strain. From the prepared microorganisms, we adjusted the density to 0.50 McFarland, which corresponds to 1.50 × 10^8^ colonies per milliliter of forming units (CFUs). The Petri dishes were inoculated with 100 μL of prepared microorganisms, and 6.0 mm blank discs (Oxoid, Basingstoke, UK) were placed on the inoculated Petri dishes. Filter paper discs impregnated with the sample of oils (10.0 μL) were placed on the agar. Subsequently, the Petri dishes were incubated for 24 h at the selected temperature: 37 °C for bacteria and 25 °C for yeasts. After 24 h, the radii of the inhibition zones formed by the oils were measured in millimeters. All strains were obtained from the Czech collection of microorganisms (Brno, Czech Republic). The experiment involved testing ten different strains of microorganisms, which included *Staphylococcus aureus* subsp. *aureus* CCM 2461, *Listeria monocytogenes* CCM 4699, *Bacillus cereus* CCM 2010, *Salmonella enterica* subsp. *enterica* CCM 3807, *Pseudomonas aeruginosa* CCM 1959, *Yersinia enterocolitica* CCM 5671, *Candida albicans* CCM 8186, *Candida glabrata* CCM 8270, *Candida krusei* CCM 8271, and *Candida tropicalis* CCM 8223. Based on the zone of inhibition, the oils were classified as having weak (0.0–5.0 mm), moderate (5.0–8.0 mm), and strong (>8.0 mm) antimicrobial activity [141,142]. The analysis was conducted in triplicate.

### 3.8. Statistical Analysis

All obtained data were statistically evaluated using the PC program GraphPad Prism 3.02 (GraphPad Software Incorporated, San Diego, CA, USA). The results are presented as the arithmetic mean of replicates together with the standard deviation (±SD) or as percentages. One-way analysis of variance (ANOVA) followed by the Tukey test was used for establishing statistical significance (*p* < 0.05) in the investigated parameters between different oils. Three independent experiments were conducted for each analysis.

## 4. Conclusions

Our comprehensive study on the effects of dried CP on the quality of commercial gourmet oils indicates that incorporating this natural additive enhances various nutritional and functional properties of the oils, thereby offering substantial health benefits. Specifically, CP influenced the FA composition, notably by increasing linoleic acid in OO and RBO and affecting all FAs in GSO. These observations can be attributed to the interactions between bioactive components of CP, the specific FAs, and antioxidant systems present in these oils. The largest share of SFAs was found in RBO-CP (22.04%), while the highest proportion of PUFAs was detected in GSO-CP (58.95%). Additionally, antioxidant activity was significantly higher in all oils flavored with CP compared to their counterparts. Olive oil with CP showed significant improvements in its antioxidant profile, enhancing its protective properties against free radicals. Despite these benefits, the addition of CP led to a significant rise in peroxide and acid values in RBO and GSO, indicating potential oxidative instability, although the induction times remained unaffected. Additionally, the antimicrobial activity of the CP-flavored oils was generally low, underscoring the need for further investigation. These findings suggest that while CP contributes to certain quality enhancements in gourmet oils, its effectiveness as a natural preservative requires further research. Future studies should focus on optimizing its formulation, such as adjusting the CP concentrations or combining it with complementary natural antioxidants, to improve the ability and bioactivity while maintaining its functional advantages. Moving forward, our next step is to evaluate the organoleptic properties of these oils using a trained sensory panel and to monitor their microbiological stability during storage. This combined approach will offer a more comprehensive understanding of both consumer perception and product safety throughout its shelf life.

## Figures and Tables

**Table 1:** The fatty acid composition (%) of evaluated edible vegetable oils.

Notation	Names of the Fatty Acids	Molecular Mass	Samples of Evaluated Edible Vegetable Oils (%)					
			RBO	GSO	OO	RBO-CP	GSO-CP	OO-CP
C14:0	Myristic acid	228.38	0.25 ± 0.00 ^b^	n.d.	n.d.	0.31 ± 0.00 ^a^	n.d.	n.d.
C16:0	Palmitic acid	256.43	16.74 ± 0.00 ^b^	8.26 ± 0.02 ^d^	11.39 ± 0.01 ^c^	18.12 ± 0.02 ^a^	8.16 ± 0.01 ^e^	11.41 ± 0.01 ^c^
C16:1	Palmitoleic acid	254.41	0.16 ± 0.00 ^c^	0.13 ± 0.00 ^d^	0.77 ± 0.00 ^a^	0.17 ± 0.00 ^b^	0.13 ± 0.00 ^d^	0.78 ± 0.00 ^a^
C18:0	Stearic acid	284.48	2.25 ± 0.01 ^d^	3.52 ± 0.00 ^a^	2.85 ± 0.00 ^b^	2.40 ± 0.00 ^c^	3.54 ± 0.01 ^a^	2.85 ± 0.00 ^b^
C18:1cis n9	Oleic acid	282.47	36.16 ± 0.02 ^c^	27.18 ± 0.00 ^d^	69.70 ± 0.01 ^a^	38.97 ± 0.01 ^b^	27.14 ± 0.01 ^d^	69.73 ± 0.07 ^a^
C18:2cis n6	Linoleic acid	280.46	41.34 ± 0.01 ^c^	57.93 ± 0.02 ^b^	11.04 ± 0.00 ^f^	36.23 ± 0.01 ^d^	58.03 ± 0.04 ^a^	11.14 ± 0.00 ^e^
C18:3 n3	*α*-linolenic acid	278.44	0.81 ± 0.00 ^c^	0.99 ± 0.00 ^a^	0.78 ± 0.00 ^d^	0.82 ± 0.00 ^c^	0.92 ± 0.00 ^b^	0.79 ± 0.00 ^d^
C20:0	Arachidic acid	312.54	0.53 ± 0.00 ^b^	0.26 ± 0.00 ^d^	0.43 ± 0.00 ^c^	0.67 ± 0.00 ^a^	0.26 ± 0.00 ^d^	0.43 ± 0.00 ^c^
C20:1 n9	cis-11-eicosenoic acid	310.52	0.33 ± 0.00 ^b^	0.23 ± 0.00 ^c^	0.30 ± 0.00 ^b^	0.41 ± 0.04 ^a^	0.20 ± 0.02 ^c^	0.31 ± 0.00 ^b^
C22:0	Behenic acid	340.60	0.18 ± 0.00 ^c^	0.27 ± 0.00^a^	0.17 ± 0.00 ^c^	0.22 ± 0.00 ^b^	0.28 ± 0.00 ^a^	0.18 ± 0.00 ^c^
C24:0	Lignoceric acid	368.64	0.24 ± 0.00 ^b^	n.d.	n.d.	0.32 ± 0.00 ^a^	n.d.	n.d.
∑ n3/n6 ratio			0.020	0.017	0.071	0.023	0.016	0.070
∑ n6/n3 ratio			50.95	58.42	14.04	44.06	63.09	14.15

Explanatory notes: RBO—rice bran oil; GSO—grape seed oil; OO—virgin olive oil; RBO-CP—rice bran oil flavored with dried Cayenne pepper red; GSO-CP—grape seed oil flavored with dried Cayenne pepper red; OO-CP—virgin olive oil flavored with dried Cayenne pepper red; n.d.—not determined; different small letters indicate statistically significant differences at the level *p* < 0.05 between oils.

**Table 2:** The peroxide and acid values, oxidative stability in edible vegetable oils.

Parameters	Samples of Evaluated Edible Vegetable Oils					
	RBO (x ± SD)	GSO (x ± SD)	OO (x ± SD)	RBO-CP (x ± SD)	GSO-CP (x ± SD)	OO-CP (x ± SD)
Peroxide value (mEq O_2_/kg)	7.20 ± 0.00 ^c^	9.20 ± 0.00 ^b^	9.00 ± 0.28 ^b^	11.60 ± 0.57 ^a^	11.20 ± 0.00 ^a^	9.40 ± 0.28 ^b^
Acid value (mg KOH/g)	0.28 ± 0.08 ^b^	0.17 ± 0.08 ^b^	0.28 ± 0.08 ^b^	1.40 ± 0.24 ^a^	1.29 ± 0.24 ^a^	0.67 ± 0.00 ^b^
Oxidative stability (h)	5.60 ± 0.36 ^b^	3.48 ± 0.10 ^c^	9.10 ± 0.28 ^a^	5.21 ± 0.18 ^b^	3.00 ± 0.06 ^c^	8.38 ± 0.00 ^a^

Explanatory notes: RBO—rice bran oil; GSO—grape seed oil; OO—virgin olive oil; RBO-CP—rice bran oil flavored with dried Cayenne pepper red; GSO-CP—grape seed oil flavored with dried Cayenne pepper red; OO-CP—virgin olive oil flavored with dried Cayenne pepper red; KOH—potassium hydroxide; x ± SD—arithmetic mean ± standard deviation; different small letters indicate statistically significant differences at the level *p* < 0.05 between oils.

**Table 3:** The antimicrobial activity of edible vegetable oils with the disc diffusion method (mm).

Microorganisms	Samples of Evaluated Edible Vegetable Oils					
	RBO	GSO	OO	RBO-CP	GSO-CP	OO-CP
	Inhibition Zone (mm)	Inhibition Zone (mm)	Inhibition Zone (mm)	Inhibition Zone (mm)	Inhibition Zone (mm)	Inhibition Zone (mm)
Gram-positive bacteria						
*Staphylococcus aureus* subsp. *aureus* CCM 2461	1.67 ± 0.58	2.33 ± 0.58	2.67 ± 0.58	2.33 ± 0.58	1.33 ± 0.58	2.33 ± 0.58
*Listeria monocytogenes* CCM 4699	2.67 ± 0.58	1.67 ± 0.58	2.67 ± 0.58	1.67 ± 0.58	1.67 ± 0.58	1.67 ± 0.58
*Bacillus cereus* CCM 2010	2.33 ± 0.58	2.33 ± 0.58	2.67 ± 0.58	2.33 ± 0.58	1.67 ± 0.58	2.33 ± 0.58
Gram-negative bacteria						
*Salmonella enterica* subsp. *enterica* CCM 3807	1.67 ± 0.58	2.67 ± 0.58	1.67 ± 0.58	2.33 ± 0.58	2.67 ± 0.58	2.67 ± 0.58
*Pseudomonas aeruginosa* CCM 1959	2.33 ± 0.58	2.33 ± 0.58	2.67 ± 0.58	1.33 ± 0.58	1.67 ± 0.58	1.67 ± 0.58
*Yersinia enterocolitica* CCM 5671	2.33 ± 0.58	2.33 ± 0.58	2.33 ± 0.58	1.67 ± 0.58	2.67 ± 0.58	1.33 ± 0.58
Yeast						
*Candida albicans* CCM 8186	2.33 ± 0.58	1.67 ± 0.58	1.67 ± 0.58	1.67 ± 0.58	1.67 ± 0.58	2.33 ± 0.58
*Candida glabrata* CCM 8270	1.67 ± 0.58	2.33 ± 0.58	1.33 ± 0.58	1.33 ± 0.58	1.33 ± 0.58	1.33 ± 0.58
*Candida krusei* CCM 8271	2.33 ± 0.58	1.67 ± 0.58	1.33 ± 0.58	1.67 ± 0.58	1.67 ± 0.58	2.33 ± 0.58
*Candida tropicalis* CCM 8223	1.33 ± 0.58	1.67 ± 0.58	1.33 ± 0.58	1.33 ± 0.58	1.33 ± 0.58	1.33 ± 0.58

Explanatory notes: RBO—rice bran oil; GSO—grape seed oil; OO—olive oil; RBO-CP—rice bran oil flavored with dried Cayenne pepper; GSO-CP—grape seed oil flavored with dried Cayenne pepper; OO-CP—olive oil flavored with dried Cayenne pepper. Based on the zone of inhibition, the oils were classified as having weak (≤5.0 mm), moderate (5.0–8.0 mm), and strong (≥8.0 mm) antimicrobial activity.

## Data Availability

The data presented in this study are available in the article.
